# The efficacy of exergaming in people with major neurocognitive disorder residing in long-term care facilities: a pilot randomized controlled trial

**DOI:** 10.1186/s13195-021-00806-7

**Published:** 2021-03-30

**Authors:** Nathalie Swinnen, Mathieu Vandenbulcke, Eling D. de Bruin, Riekje Akkerman, Brendon Stubbs, Joseph Firth, Davy Vancampfort

**Affiliations:** 1 KU Leuven Department of Rehabilitation Sciences, Leuven, Belgium; 2 University Psychiatric Centre KU Leuven, Leuven, Kortenberg Belgium; 3 KU Leuven Department of Neurosciences, Leuven, Belgium; 4 Department of Health Sciences and Technology, Institute of Human Movement Sciences and Sport, ETH Zürich, Leopold-Ruzicka-Weg 4, 8093 Zürich, Switzerland; 5 Division of Physiotherapy, Department of Neurobiology, Care Sciences and Society, Karolinska Institute, Stockholm, Sweden; 6 Long-term care facility De Wingerd, Leuven, Belgium; 7 South London and Maudsley NHS Foundation Trust, Denmark Hill, London, UK; 8 Institute of Psychiatry, Psychology and Neuroscience (IoPPN), King’s College London, London, UK; 9 Division of Psychology and Mental Health, Faculty of Biology, Medicine and Health, University of Manchester, Manchester, UK; 10 NICM Health Research Institute, Western Sydney University, Sydney, New South Wales Australia

**Keywords:** Brain plasticity, Cognition, Dementia, Depression, Physical activity, Physical fitness, Serious exergames

## Abstract

**Background:**

It is currently unknown whether exergaming is efficacious in people with major neurocognitive disorder (MNCD) residing in long-term care facilities. This pilot randomized controlled trial (RCT) explored the efficacy of a stepping exergame program on gait speed, balance, mobility, reaction time, cognitive and neuropsychiatric outcomes, quality of life, and daily life functioning in people with MNCD residing in long-term care facilities.

**Methods:**

Participants were randomly assigned to 8 weeks, three times weekly, 15 min of exergaming versus watching preferred music videos. The exergame device consisted of a pressure-sensitive step training platform on which participants performed stepping movements to play the games. The device automatically adapted the training level to the participants’ capabilities. The Short Physical Performance Battery (SPPB), step reaction time test (SRTT), Montréal Cognitive Assessment (MoCA), Neuropsychiatric Inventory (NPI), Cornell Scale for Depression in Dementia (CSDD), Dementia Quality of Life (DQoL), and Katz Activities of Daily Living (Katz ADL) were assessed at baseline and post-intervention. A Quade’s non-parametric ANCOVA controlling for baseline values with post hoc Bonferroni correction (*p* < 0.00625) was used to analyze pre- and post-differences between the groups. Partial eta-squared (η^2^p) effect sizes were calculated.

**Results:**

Forty-five of 55 randomized inpatients with mild to moderate MNCD (Mini-Mental State Examination score = 17.2 ± 4.5; aged 70–91; 35 women) completed the study. The exergame group (*n* = 23) demonstrated improvements in gait speed (*p* < 0.001, *η*^*2*^_*p*_ = 0.41), total SPPB (*p* < 0.001, *η*^*2*^_*p*_ = 0.64), SRTT (*p*<0.001, *η*^*2*^_*p*_ *=* 0.51), MoCA (*p*<0.001, *η*^*2*^_*p*_ *=* 0.38), and reductions in CSDD (*p*<0.001, *η*^*2*^_*p*_ *=* 0.43) compared to the control group (*n* = 22). There were no differences in NPI (*p* = 0.165, *η*^*2*^_*p*_ *=* 0.05), DQoL (*p* = 0.012, *η*^*2*^_*p*_ *=* 0.16), and ADL (*p* = 0.008, *η*^*2*^_*p*_ *=* 0.16) post-intervention scores between the experimental and control group, albeit DQol and ADL measures showed large effect sizes in the exergame group. The mean attendance rate was 82.9% in the exergame group and 73.7% in the music control group. There were no study-related adverse events reported by the participants, nor observed by the research team.

**Conclusions:**

The findings of this pilot RCT suggest that an individually adapted exergame training improves lower extremity functioning, cognitive functioning and step reaction time and symptoms of depression in inpatients with MNCD residing in long-term care facilities.

**Trial registration:**

ClinicalTrials.gov, NCT04436302

## Introduction

Major neurocognitive disorder (MNCD) is a syndrome characterized by cognitive function impairment, motor decline, and psychological and behavioral problems [1]. In 2019, there were worldwide over 50 million people with MNCD. This number is estimated to increase to 152 million by 2050, predominantly driven by population aging [2]. The progressive functional decline observed in people with MNCD contributes to a reduced quality of life, loss of independence, and increased caregiver effort [3]. Higher levels of care often result in transfers to long-term care facilities [4]. This is placing a substantial burden on health care systems and has resulted in MNCD being considered a public health priority by the World Health Organization [5].

No disease-modifying treatments, pharmacological nor non-pharmacological, have been developed that can cure MNCD or halt its progression [6]. Therefore, the main goals of caring for people with MNCD admitted to long-term care facilities are maintaining or improving their physical condition and their mental and physical quality of life. To this end, non-pharmacological therapies targeting people’s lifestyle are recommended as a first-line approach [7]. Potential strategies to improve health and well-being of people with MNCD in long-term care facilities include environment adaptation [8], sensory interventions, and physical activity [9]. Physical activity can delay the course of the disease and thereby counteract the decline in functionality [9, 10]. In addition, physical activity improves gait speed, balance, mood, and execution of daily life activities [9, 11]. Improvement in gait speed is particularly relevant as slow gait speed is associated with an increased risk of falls in people with MNCD [12], which on its turn is associated with higher rates of co-morbidity and premature mortality [13].

Despite the benefits, engaging people with MNCD residing in long-term care facilities in physical activity is often a challenge. Behavioral symptoms like agitation and passivity which are exhibited by 90% of nursing home residents with MNCD [14] and disorientation and decreased interest [15] are the most important barriers. The physical activity levels of residents decrease steadily after admission to a long-term care facility [16] and people with MNCD who are residing in a nursing home environment remain physically inactive for most of the day [8].

Innovative developments in technology have provided new options to engage older adults with MNCD in an enjoyable way through exergames [17]. Exergames combine physical activity and cognitive tasks [18, 19] and require the player to produce body movements in response to visual, auditory, and somatosensory cues [20]. A recent qualitative study demonstrated that people with MNCD residing in long-term care facilities enjoy exergaming [21]. In this study, the mean attendance rate in 24 sessions during 8 weeks at three times per week was 79.3%. In recent years, a growing interest has emerged as well in the efficacy of exergaming in people with MNCD [15, 17, 22–24]. A previous meta-analysis demonstrated that in residential and community-dwelling older adults with mild cognitive impairment or MNCD, a combination of cognitive and physical training resulted in small-to-medium improvements in global cognitive function and mood and moderate-to-large positive effects on activities of daily living [10]. A randomized controlled trial in outpatients and community patients with MNCD suggested that the group receiving combined cognitive–aerobic bicycle exergame training during 30 to 50 min at three times per week during 12 weeks and the group receiving cycling at the same frequency, intensity, and time without exergaming improved psychomotor speed [25] and reduced frailty [26], as compared to the control group, receiving relaxation and flexibility exercises. However, no differences were observed between the three groups for executive functioning, episodic memory and working memory [25, 26].

To the best of our knowledge, no randomized controlled trial (RCT) has previously explored the efficacy of a stepping exergame program on physical fitness parameters including gait speed, mobility and balance, step reaction time, neuropsychiatric symptoms, mood, and quality of life in people with MNCD. Moreover, it is currently unknown whether exergaming is also efficacious in people with MNCD residing in long-term care facilities. A qualitative study in people with MNCD in long-term care facilities did show promising effects of exergaming on self-reported cognitive functioning, activities of daily living performance, fear of falls, quality of life, mood, and several physical outcomes, such as gait speed, mobility, and balance [21]. However, these preliminary qualitative findings need to be confirmed in a RCT.

Therefore, the goal of the current pilot RCT was to fill these gaps in the literature by exploring the physical, mental, and cognitive effects of an exergame program added to care as usual as compared to a passive control condition (i.e., watching preferred music videos) in people with MNCD residing in long-term care facilities.

## Methods

### Participants and procedure

This pilot RCT was conducted following the Consolidated Standards of Reporting Trials guidelines (CONSORT, Boutron et al., 2008; Cuschieri, 2019). The CONSORT extensions for pilot abstract and pilot trials were added in Additional file 1 [27]. The trial was registered in ClinicalTrials.gov (Identifier: NCT04436302). Inpatients of the University Psychiatric Centre KUL in Kortenberg and residents of long-term care facility de Wingerd in Leuven (Vlaams Brabant, Belgium) diagnosed with MNCD were included. Participants were randomly assigned by an independent statistician using a random number generator (https://www.random.org/) to either 8 weeks, three times per week 15 min of exergaming (experimental group), added to care as usual, or seated listening to favorite music (control group), at a same volume, added to care as usual. Care as usual consisted of pharmacotherapy and physiotherapy focusing on comfort care. Participants were assessed at baseline (pretest) and after 8 weeks (posttest) by a physiotherapist blinded to group allocation using the Short Physical Performance Battery (SPPB) in order to assess gait speed, balance and lower limb strength [28, 29], a step reaction time test, the Montréal Cognitive Assessment (MoCA) [30–32], the Neuropsychiatric Inventory (NPI) [33], the Cornell Scale for Depression in Dementia [34], the Dementia Quality of Life (DQoL) questionnaire [35, 36], and Activities of Daily Living [37, 38]. The study protocol was approved by the Medical Ethics committee of UZ Leuven (reference number S60641). All participants and their legal representatives gave their written informed consent. No compensation was granted to the participants.

### Power analysis

The principal outcome measure was gait speed. An exergame training program including balance training demonstrated that gait speed can be increased by 0.13 m per second [39] and a meaningful change lies around 0.10–0.15 m per second [39–41]. Older adults in long-term care in general exhibit on average a walking velocity of 0.475 m per second based on the SPPB [42]. In older adults in long-term care, a sample size of 12 participants per group enables detection of a within-group difference, from baseline to post-test, of 0.105 m per second (SD = 0.16) on gait speed when a medium-sized effect is expected [43, 44]. However, we decided to follow the guidelines of Whitehead et al., who recommend a sample size of at least 16 but ideally 26 participants per group for a medium effect size in pilot trials [45]. To account for attrition over time, 15% can be expected with RCTs of exercise programs in long-term care facilities [46]. The required a-priori sample size was increased by 20% to minimum 20 but ideally 30 participants per group.

### Eligibility criteria

Inclusion criteria were as follows: (a) a Diagnostic and Statistical Manual of Mental Disorders (DSM) 5 diagnosis of MNCD (American Psychiatric Association, 2013), (b) aged 65 years or older, (c) a score of minimum 10 on the Mini-Mental State Examination (MMSE) [47] as assessed by the research team, (d) having been residing at least two weeks in the care facility at the time of inclusion and with a perspective of at least 10 more weeks of stay, (e) and being physically capable of doing standing exercises (whether or not with extra support). Possible causes of MNCD were vascular dementia, Alzheimer’s disease, mixed dementia, Parkinson’s disease, frontotemporal degeneration, or Lewy body disease, as diagnosed by the treating psychiatrist. Exclusion criteria consisted of the following: (a) any unstable cardiovascular or other health condition which, according to the American College of Sports Medicine Standards, might lead to unsafe participation, (b) a score lower than 10 on the MMSE, and (c) a planned transfer to another setting within the following 2 months.

### Experimental condition

**Fig. 1:**
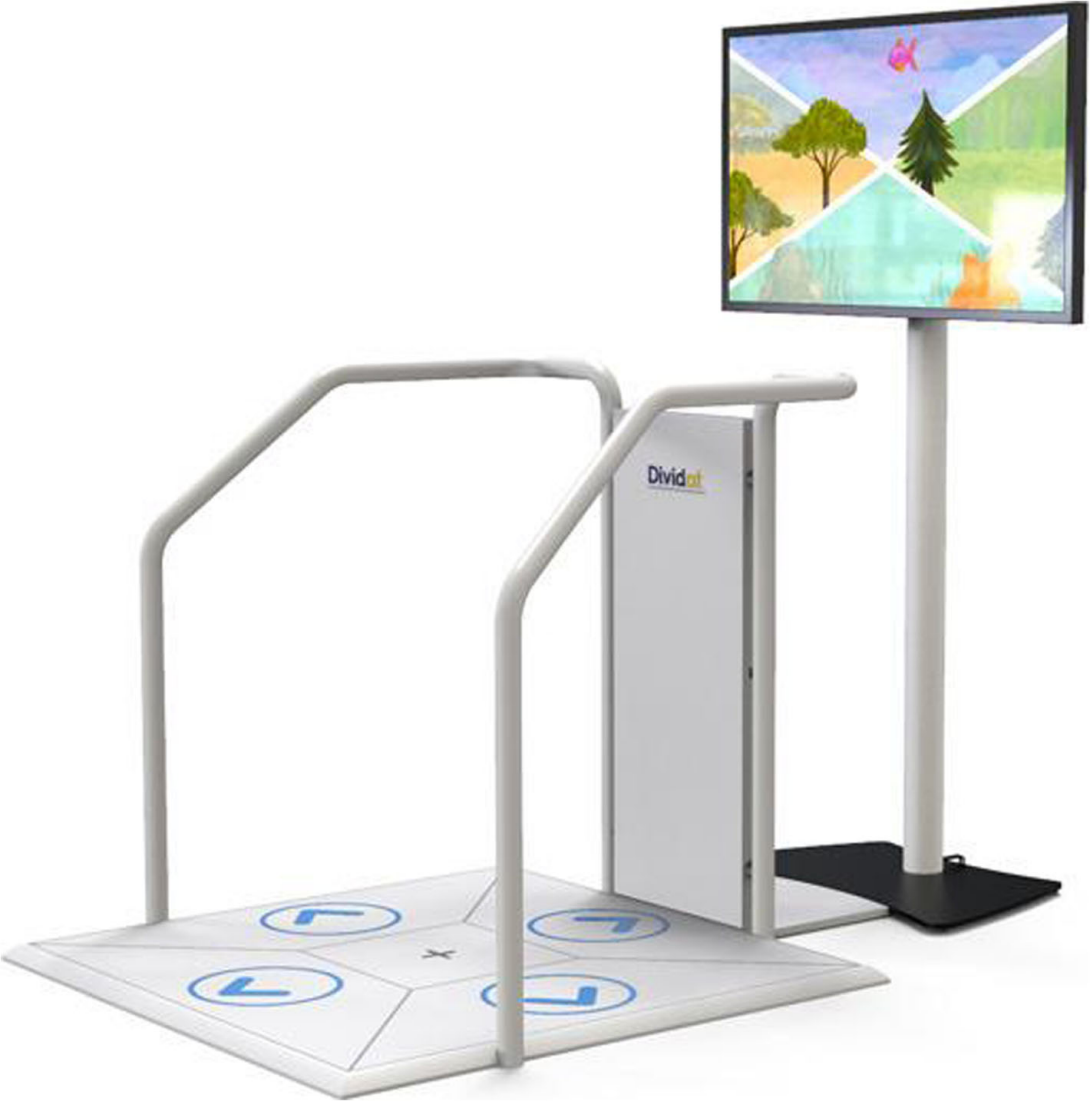
Dividat Senso exergame device. Permission was obtained from the copyright holder. Picture from the website dividat.com

Participants performed three individual sessions per week for a period of 8 weeks, resulting in a total of maximum 24 sessions. Each session consisted of a walk to the exercise room (i.e., approximately 10 min), 15 min of exergaming, and a walk back to the ward. The duration of exergaming in previous research varied from 10 min [48, 49] up to 60 min [50]. A duration of 15 min was expected to be feasible and not too demanding for this specific population. The exergame device “Dividat Senso” (Dividat, Schindellegi, Switzerland) was administered. This device consisted of a step training platform (1.13 m × 1.13 m) which was sensitive to pressure changes (strain gauges measuring at 50 Hz). The sensors detected steps in four directions: left, right, top, and bottom. Participants could grasp the bars on waist height, when deemed necessary. Participants that used a wheelchair were assisted by the guiding therapist to stand up and walk to the Dividat Senso exergame device. The platform was connected via a USB cable to a computer and a frontal television screen (LG, 94.5 cm × 53 cm, model 43LJ500V, 43 in.) on which the exergames were displayed. A picture of the Dividat Senso exergame device can be found in Fig. 1. The starting position was an upright stance with both feet in the middle of the platform. Participants interacted with the game interface by pushing one foot on one of the four different arrows. When the game required the player to perform a step to the left or right, the associated lower limb was used. For a step in the two other directions, the player used a lower limb of preference. The games trained the following cognitive abilities: divided and selective attention, flexibility, postural control, and visuospatial working memory. The device provided real-time visual, auditory and somatosensory (vibrating platform) cues, and feedback in order to enrich the game experience. The sessions consisted of multiple games and the duration of each video game varied between 120 and 200 s. The following training principles [51] were implemented: (a) task difficulty was individually adapted to facilitate retention, (b) training variability was provided in order to enhance task transfer, (c) a system of feedback to improve training effects, and (d) individual difficulty zones to match the training level of each participant. The physical therapist designed an individual program for each participant, adapted to the participants’ functionality, cognition, and health status. The exergames automatically adapted to the participants’ capabilities during active exergaming, i.e., providing more difficult stimuli when the players reacted fast and correct. During the 8-week program, progress was also made regarding the reduction of manual support via the bars. All participants were individually supervised to ensure safety and comfort. A Template for Intervention Description and Replication (TIDieR) checklist [52] was added in Additional file 2. Also, a description of the specific games and their characteristics are included in Additional file 3.

### Control condition

Participants watched and listened 15 min, three times per week for 8 weeks to preferred music videos on a television screen (LG, 94.5 cm × 53 cm, model 43LJ500V, 43 in.) in the same exercise room. Each session consisted of a walk to the exercise room (i.e., approximately 10 min), 15 min of watching and listening to music videos, and a walk back to the ward. All participants were individually supervised to promote comfort and personal interaction with the therapist, who was the same person as the therapist in the experimental condition. Listening to music is a non-aerobic intervention which is recommended in people with MNCD [53].

### Outcome measures

#### Short Physical Performance Battery (SPPB)

Gait speed, balance, and lower limb strength were assessed with the SPPB [28, 29]. It is composed of three subtests; a hierarchical standing balance test, a short 4-m walk at usual pace [54], and 5 chair rises. The maximal total score is 12 and higher total scores indicate a better lower extremity functioning. Total scores between 10 and 12 indicate good functioning and no risk of developing mobility disability, total scores between 4 and 9 indicate an elevated risk, and scores between 0 and 3 indicate an already present loss of mobility. The minimal detectable change (MDC) of our primary outcome, gait speed, on the 4-m walk test is 0.28 m per second [55]. Although researchers have previously expressed concerns regarding the feasibility and validity of quantitative functional assessments in people with MNCD [56, 57], the reliability of the SPPB is high in older adults with and without MNCD, with intraclass correlation coefficient (ICC) values ranging between 0.82 and 0.92 [55, 58, 59]. Test instructions were concise and repeated when needed, and tasks were presented by the guiding therapist [56]. Participants were allowed to use their assistive devices such as a walker or a walking cane.

#### Step reaction time test (SRTT)

Average choice step reaction time for all four directions together was assessed by means of the “Simple” protocol of the Dividat Senso device. Four white circles were displayed on the television screen. These circles corresponded to the four arrows on the step platform (up, down, left, and right). The step direction was indicated by one of the four circles turning red. Participants were instructed to step as quickly as possible onto the corresponding arrow of the step platform and to return to the center. The program was designed so that the number of stimuli and the speed at which the stimuli were presented accelerated in line with game performance. In other words, the game adapted to the individuals’ performance and was therefore not standardized. Choice step reaction time was measured from stimulus occurrence to step finalization and was expressed in milliseconds.

Although this test has not been psychometrically examined, choice reaction time tests have been developed and examined in the past. A simple test for unplanned volitional steps has excellent predictive validity for future falls, good inter-day test-retest reliability (ICC 0.74) and excellent criterion validity with a strong bivariate correlation between a simple “low-tech” choice stepping reaction time test and the electronic version of the choice stepping reaction time test (*r* = 0.81, *p* < 0.001) [60, 61].

#### Montréal Cognitive Assessment (MoCA)

The MoCA, which is designed for milder forms of MNCD, assesses memory, language, executive functions, visuospatial skills, attention, concentration, abstraction, calculation, and orientation using a paper and pencil test. The MoCA has a good construct validity (*r* values range from 0.46 to 0.75) [62], inter-rater reliability (*r* = 0.97), test-retest reliability (*r* = 0.88), and internal consistency (Cronbach’s alpha = 0.89) [63]. We used the total score (ranging from 0 to 30) with higher scores indicating better cognitive functioning.

#### Neuropsychiatric Inventory (NPI)

Psychopathological problems were assessed with the NPI [33]. It was administered in a semi-structured interview setting with a close caregiver. One screening question per behavior domain was asked to the caregiver, after which approximately seven in-depth questions were possible. The behavior domains are delusions, apathy, hallucinations, disinhibition, agitation/aggression, irritability, depression/dysphoria, aberrant motor behavior, anxiety, nighttime behavior disturbances, euphoria, and appetite and eating abnormalities. Behavior frequency is scored on a 4-point scale, ranging from 1 to 4. Symptom severity is scored on a 3-point scale ranging from 1 to 3. The NPI total score is calculated by multiplying the frequency and severity rates per domain and adding them up. The total score ranges from 0 to 144. The test–retest reliability is 0.79 for behavior frequency (*P* = 0.0001) and 0.86 for symptom severity (*P* = 0.0001) [64]. The Cronbach’s alpha coefficient for the overall score is 0.88 [65].

#### Cornell Scale for Depression in Dementia (CSDD)

Symptoms of depression were assessed with the observation-based CSDD, in the form of an interview with the caregiver. The CSDD consists of 19 items and each one is scored on a 3-point scale ranging from 0 to 2 [34]. Items are clustered into five categories: mood, behavioral disturbance, physical signs, cyclic functions, and ideational disturbance. The CSDD has adequate internal consistency and reliability in an older, frail nursing home population with MNCD. Cronbach’s alpha is 0.81 and the kappa values of two studies included are 0.57 and 0.91 [66].

#### Dementia Quality of Life (DQoL) questionnaire

Quality of life was assessed with the DQoL questionnaire [36], which is widely used in clinical practice. It consists of 29 items, measuring five domains: self-esteem (4 items), positive affect (6 items), negative affect (11 items), feelings of belonging (3 items), and sense of esthetics (5 items). A higher score per domain reflects better QoL, except on the negative affect dimension. Cronbach’s alpha coefficient for internal consistency ranges from 0.71 to 0.84. The ICC for test-retest reliability ranges from 0.69 to 0.80 [35].

#### Activities of Daily Living

The interviewer-administered Katz ADL Index [38] was used to evaluate participants’ function in terms of level of independence or dependence when performing certain daily living activities. It consists of six items: bathing, dressing, transfers, toileting, continence, and feeding. Each item is scored from 1 (independent) to 4 (dependent). The internal consistency of the Katz ADL Index is good with a Cronbach’s alpha of 0.96, despite the existence of cognitive decline [67].

#### Secondary outcomes

Secondary outcome measures were attendance and adherence rates. Attendance sheets were completed during each session to record the number of training sessions. Attendance rates were measured by dividing the number of attended training sessions by the maximum possible number of training sessions (24 sessions). An attendance rate of 70% or higher (minimum 17 attended out of 24 planned sessions) was deemed as being adherent to the exergame program [68]. The adherence rate was determined by calculating the dropouts (attrition) as a percentage of the entire sample size. Participants signed an informed consent stating that they were not obliged to give a reason for non-attendance or drop-out. Therefore, it was not possible to calculate reasons for non-attendance.

### Statistical analysis

Data were screened for normality using the Shapiro-Wilk test. Since outcome data were not normally distributed, the between-group post-outcome differences were analyzed with Quade’s non-parametric analysis of covariance (ANCOVA). Although there were no significant differences in baseline values between the experimental and control group, we corrected for the baseline values. However, in order to be able to compare the outcome data with other studies, data are presented as mean ± standard deviation. Imputation methods for missing data were not applied given evidence of the subsequent risk of bias [69]. Partial eta-squared (η^2^p) effect sizes were calculated where a η^2^p of 0.01 to < 0.06 was considered small, 0.06 to < 0.14 medium, and 0.14 or higher considered as a large effect size [70]. We corrected for multiple testing. The Bonferroni-corrected statistical significance level was set at *P* < 0.00625 (0.05/8 comparisons). Differences in demographic characteristics between the experimental and control groups were tested using the Mann-Whitney *U* test. Differences in categorical variables were tested using Fisher’s exact test. Data were analyzed with SPSS Version 26 (Armonk, NY, USA, 2017).

## Results

### Participants

**Fig. 2:**
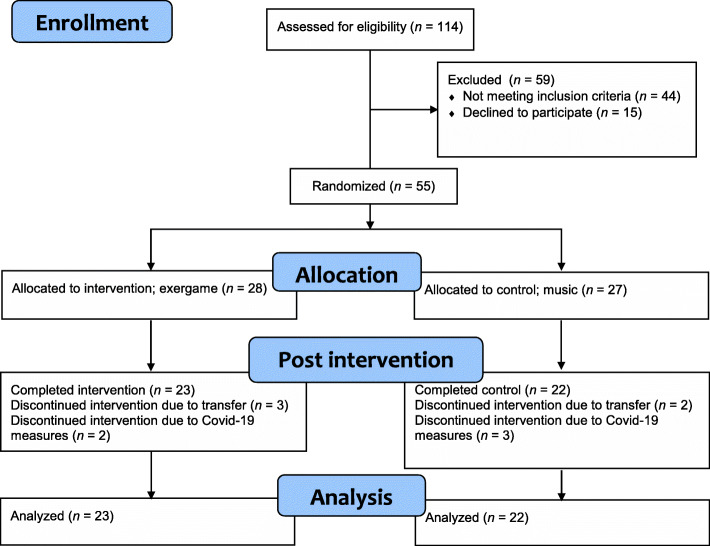
CONSORT diagram of participant flow

A total of 114 participants were assessed for eligibility, of which 44 failed to meet the inclusion criteria. Another 15 people refused to participate. Of this group, 4 (26.7%) did not give a specific reason, ten (66.7%) were not interested, and one (6.7%) performed the training program but withdrew consent following the intervention. Finally, 45 participants (11 from the University Psychiatric Centre KUL and 34 from long-term care facility de Wingerd) consented to participate in this study and were randomly assigned to either the exergame intervention (*n* = 28) or music control group (*n* = 27). Five subjects dropped out due to transfer to another setting. Another five were excluded as the study ended prematurely due to COVID-19 measures. In total, 23 participants in the exergame group and 22 in the control group completed the study. The flow chart is illustrated in Fig. 2.

A more detailed description of the participants’ individual characteristics is provided in Table 1. Thirty-five women (77.8%) and 10 men (22.2%) were included, with a mean age of 85.0 (SD = 6.0) and mean MMSE score of 17.2 (SD = 4.5). Information on diagnosis and somatic comorbidities were taken from patient charts. Eleven participants (24.4%) used an assistive walking device.

**Table 1:** Participant characteristics

Variables	Sub-category	Exergame group (***n*** = 23)	Music group (***n*** = 22)	***p***^a^
Age in years, mean (SD)		84.7 (5.6)	85.3 (6.5)	0.224
Gender, *n* (%)	Male	5 (21.7)	5 (22.7)	> 0.999
	Female	18 (78.3)	17 (77.3)	> 0.999
MMSE, mean (SD)^b^		18 (4.4)	17 (4.2)	0.102
Diagnosis, *n* (%)	Alzheimer’s disease	17 (73.9)	14 (63.6)	0.530
	Vascular dementia	2 (8.7)	4 (18.2)	0.414
	Mixed Alzheimer’s and vascular dementia	2 (8.7)	3 (13.6)	0.665
	Frontotemporal Degeneration	0 (0)	1 (4.5)	0.489
	Lewy body disease	0 (0)	0 (0)	> 0.999
	Neurocognitive disorder not otherwise specified	2 (8.7)	0 (0)	0.489
Somatic comorbidities, *n* (%)	Diabetes type II	2 (8.7)	5 (22.7)	0.243
	Heart disease	8 (34.8)	11 (50.0)	0.375
	Hypertension	4 (17.4)	0 (0)	0.109
	Gait disorders	12 (52.2)	14 (63.6)	0.550
	Visual deficiencies (cataract, glaucoma)	3 (13.0)	0 (0)	0.233
Indoor mobility, *n* (%)	Wheelchair	2 (8.7)	0 (0)	0.49
	4-wheeled walker	3 (13.0)	4 (18.2)	0.70
	Single-point walking cane	0 (0)	2 (9.1)	0.23
	No walking aid	18 (78.3)	16 (72.7)	0.74
Attendance rates (%)		82.9	73.7	0.07

^a^*p* values of group comparisons refer to Mann-Whitney *U* tests for continuous variables and Fisher’s exact tests for categorical variables

^b^Scores on the MMSE range from 0 (severe impairment) to 30 (no impairment)

*MMSE* Mini-Mental State Examination

There was no difference in age between both groups (*p* = 0.224) nor a difference in gender distribution (*p* = 0.609) or baseline MMSE (*p* = 0.102).

### Effects of exergame intervention

The means and standard deviations of the pretest and posttest battery scores for the intervention and control groups and the *P* values, *F* values, and partial eta squared (*η*^*2*^_*p*_) are depicted in Table 2. The medians and interquartile ranges can be found in Additional file 4: Supplementary Table 1. Compared to the control group, the exergaming group had significantly better gait speed after the intervention (*p* < 0.001, *η*^*2*^_*p*_ = 0.410). Significantly more participants in the exergame group improved beyond the minimal clinical detectable change on gait speed than participants in the music control condition (47.8% vs. 0%, *p* < 0.001). Participants in the exergame group also had an overall better lower extremity functioning as assessed with the SPPB (*η*^*2*^_*p*_ = 0.643, *p* < 0.001) post-intervention. SRTT scores (*η*^*2*^_*p*_ *=* 0.506, *p* < 0.001) and MoCA scores (*η*^*2*^_*p*_ *=* 0.385, *p* = < 0.001) also improved significantly after 8 weeks of exergame training. In addition, CSDD scores improved significantly after 8 weeks of exergame training (*η*^*2*^_*p*_ *=* 0.431, *p* < 0.001). There were no significant differences in NPI (*η*^*2*^_*p*_ *=* 0.051, *p* = 0.165), DQoL (*η*^*2*^_*p*_ *=* 0.157, *p* = 0.012) and ADL (*η*^*2*^_*p*_ *=* 0.161, *p* = 0.008) post-intervention scores between the experimental and control group, albeit DQoL and ADL measures showed large effect sizes.

**Table 2:** The effects of an exergame intervention and a music intervention on measured outcomes

Variable	Intervention (***n*** = 23)		Control (***n*** = 22)		***P***	***F***	***η***^***2***^_***p***_
	Pre test (mean ± SD)	Post test (mean ± SD)	Pre test (mean ± SD)	Post test (mean ± SD)			
SPPB	5.5 ± 1.9	8.5 ± 2.5	5.2 ± 2.9	3.8 ± 2.5	< 0.001*	72.1	0.64
Gait speed (m/s)	0.6 ± 0.2	0.8 ± 0.3	0.6 ± 0.2	0.5 ± 0.2	< 0.001*	29.3	0.41
SRTT (ms)	2827.1 ± 1884.1	1426.6 ± 333.1	4551.1 ± 4243.6	5292.9 ± 4893.4	< 0.001*	38.8	0.51
MoCA	9.4 ± 4.1	12.1 ± 5.2	8.5 ± 5.2	5.7 ± 4.0	< 0.001*	24.4	0.38
NPI	11.4 ± 12.8	6.6 ± 11.4	8.4 ± 7.4	16.1 ± 15.1	0.165	2.0	0.05
CSDD	7.0 ± 6.4	3.0 ± 4.4	5.3 ± 4.5	9.3 ± 6.7	< 0.001*	28.8	0.43
DQoL	2.7 ± 0.7	3.5 ± 0.9	3.0 ± 0.9	3.0 ± 0.9	0.012	6.9	0.16
ADL	9.0 ± 2.1	9.0 ± 2.3	10.1 ± 2.8	11.4 ± 3.4	0.008	7.7	0.16

*Significant when *P* < 0.00625 (0.05/8 comparisons) using Quade’s non-parametric analyses of covariance with post test scores as dependent variables, groups as independent variables, and baseline scores as covariates. ADL, activities of daily living (range = 6 to 24 with higher scores indicating higher dependency in activities of daily living); CSDD, Cornell scale for depression in dementia (range = 0 to 38, and a score below 6 is associated with absence of depressive symptoms, and scores above 10 indicate probable major depression); DQoL, Dementia Quality of Life (scores range from 1 (poor QoL) to 5 (excellent QoL)); MoCA, Montréal Cognitive Assessment (total scores range from 0 to 30 with lower scores indicating more cognitive impairment); NPI, Neuropsychiatric Inventory (12-item score with a range of 0 to 12 per item); SPPB, Short Physical Performance Battery (total scores range from 0 to 12 with lower scores indicating a higher risk and a score lower than 10 indicates one or more mobility limitations); SRTT, step reaction time test (lower values indicate a faster reaction time)

### Attendance, adherence, and safety

The mean attendance rate was 82.9% (SD = 13.14) in the exergame intervention group and 73.7% (SD = 25.20) in the music control group. None of the attended sessions, both in the exergame and in the music condition, had to be stopped prematurely. Nineteen participants in the exergame intervention group and 13 participants in the music control group reached an attendance rate of at least 70% of the sessions, which was deemed as being adherent to the exergame program [68]. The self-reported training intensity varied from mild to moderate. There were no study-related adverse events reported by the participants, nor observed by the research team.

## Discussion

To the best of our knowledge, this is the first RCT to investigate the physical, mental, and cognitive effects of 8 weeks of stepping exergame training, added to care as usual, in institutionalized people with MNCD. Compared to adding non-aerobic music video listening and watching (for a duration of 8 weeks, three times per week for 15 min), involvement in stepping exergame training (three times per week for 15 min during 8 weeks) in addition to care as usual significantly improved gait speed, mobility, and balance as measured with the SPPB, step reaction time as measured with the SRTT, and cognitive function as measured with the MoCA. In addition, symptoms of depression, as measured with the CSDD, were significantly reduced in the latter group. No significant effects were found on neuropsychiatric symptoms as measured with the NPI, quality of life as measured with the DQoL questionnaire, and ADL functioning as assessed with the Katz ADL Index, albeit DQoL and ADL measures showed large effect sizes.

Our finding that exergaming improved lower extremity functioning is in contrast with previous cognitive-aerobic bicycle training trials in community-dwelling people with mild MNCD [25, 71]. A possible reason for the difference with previous bicycle training research is that our participants performed standing balance exergames that required participants to stand and perform stepping movements, which directly addresses gait and balance [72]. The exergame we used utilized a changing base of support required to play the games which better meets the specifics for training postural control [73] as compared to sitting on a stationary bicycle. The contrasting findings may also be explained by the spatial versus non-spatial processing demands in combination with the postural modality of the exercises [74]. Whereas exercising in an upright body position enhances both processing speed and attentional selectivity [75], such effects are not observed for exercise performed pedaling a bicycle in a seated position, indicating that body posture during exercise has a dynamic influence on visual working memory performance [74]. A previous pilot study investigating a Wii-Fit program and walking program, both performed for 30 min daily, five times a week for 8 weeks by participants with mild Alzheimer’s disease living in an assisted living facility, also resulted in comparable improvements in gait and balance in both groups, as measured by the Berg Balance Scale and the Tinetti test [76]. Similarly, an eight-week home-based Wii-Fit training for 30 min daily, five times a week in community-dwelling people with mild Alzheimer’s disease resulted in improvements in BBS scores, which were sustained after 16 weeks of follow-up, as compared to a walking group with comparable training duration [77]. The observed improvements in SPPB scores in the present study are considered of high clinical relevance as low scores on the SPPB including a low gait speed are highly predictive of health-related consequences, such as mobility impairment [78], future hospitalization [79], longer hospitalization duration [80], admission to long-term care facilities, premature mortality [28, 81], and falls [82]. Improved gait speed is also known to be associated with a faster step reaction time, and both an improved gait speed and faster step reaction time are associated with a reduced fall risk [12, 54]. The current findings do show that exergaming improves the step reaction time significantly as well. It should however be noted that the modality of the SRTT was similar to one of the exergames. A learning effect therefore cannot be excluded in the intervention group compared to the control group.

Besides improvements in physical parameters, cognitive functioning, as assessed with the MoCA, ameliorated. These findings are in line with a previous meta-analysis of RCTs demonstrating that in older adults with MCI or MNCD, a combined cognitive-physical training program leads to small-to-medium improvements in cognitive function [10].

A final important observation was that our exergaming program induced significant reductions in depression, as measured with the CSDD following exergaming versus music video listening and watching. A recent systematic review of exergames in older adults found these interventions to be generally effective for reducing depression, particularly in older adults with high depressive symptoms [83].

Despite improvements in QoL, these changes were not significantly different from the control condition. A reason might be that music interventions are known to improve QoL in this population [53]. Another reason might be that we investigated individual exergame training. Previous research in people with MNCD showed that in particular exergaming in a group improves QoL [84].

Although not significant, we observed large effect sizes for improvements in ADL. The lack of significant improvements in ADL is in line with a study examining the effects of a Nintendo-Wii bowling game in residents of retirement homes with and without MNCD [50]. In a more recent RCT, no improvements in Katz ADL were found after 12 weeks of cognitive-aerobic bicycle training on a stationary bike in community-dwelling older adults with MNCD [26]. The findings in our study and previous RCTs in MNCD are, however, not consistent with a meta-analysis exploring the efficacy of combined cognitive-physical training in older adults with MCI or MNCD that demonstrated moderate-to-large positive effects on activities of daily living [10]. However, this meta-analysis also included people with MCI with less severe ADL impairments, which limits comparability with our results.

A strength of the study is that we also included older adults with MNCD using canes, walkers, and wheelchairs. Therefore, our findings are generalizable to mobility-impaired people with MNCD. All participants were able to acquire and retain motor skills in the exergame environment. The notable adherence rates and absence of reported adverse events are additional strong features of our intervention. These findings suggest that individual exergame sessions, supervised one on one by a physical therapist, are feasible and efficacious in this vulnerable population.

Future research using an exergame approach is warranted and should explore the underlying mechanisms of the observed benefits. For example, it is known that exercise leads to elevated levels of brain-derived neurotrophic factor, which supports the growth and maintenance of neurons. Hippocampal structure and function are altered by exercise and memory and cognitive function are facilitated [85]. It is also possible that the observed improvements in executive functions in our study might be due to modulations in prefrontal cortex oxygenation [86] or to the reorganization of neuronal networks [87]. However, larger trials are needed to investigate these mechanisms in more detail. Moreover, there is increasing evidence that the combination of physical and cognitive activity may have synergistic effects [88, 89]. While physical exercise facilitates plasticity, cognitive activity guides the plastic changes [90]. Future research could, for example, compare the effects and dose-response of exergaming versus aerobic and/or strength training and/or balance training on physical, mental, and cognitive outcomes and underlying mechanisms in people with MNCD. Other opportunities for future research include further exploration of the physical, mental, and cognitive effects of exergaming using mixed methods randomized controlled designs where the quantitative outcomes can be enriched by the participants’ subjective perceptions. Future research should explore the efficacy of exergaming on gait and balance outcomes to explore possible exercise interventions for this population. Long-term follow-up of the effects of exergame training on the examined outcomes is warranted to explore possible maintenance effects. Furthermore, incidence of falls, fall-related injuries, and fall efficacy should also be explored, in particular since it has been demonstrated that step training can prevent falls by 50% in older adults in both community and institutional settings [91]. Large scale RCTs should also explore whether the beneficial effects might differ between different diagnoses of MNCD and between different levels of MNCD severity and could investigate differences in outcomes between individual training versus group training interventions. Finally, implementation research is needed to explore the (cost-)effectiveness of exergaming in people with MNCD in psychiatric and residential care. Such trials are ongoing [92].

### Limitations

Although very promising, the findings of the current RCT need to be interpreted with caution due to some limitations. First, this was a pilot RCT exploring the efficacy of exergaming in a wide range of physical, cognitive, and mental parameters in people with MNCD residing in residential care settings. Although our pilot trial had sufficient power, larger trials comparing exergaming with other active control interventions such as aerobic exercise need to confirm or refute our preliminary findings. Second, our study was limited to solely two care institutes in one geographical area in Belgium which limits generalizability to other settings and countries. Third, since only inpatients who were motivated to participate in the exergame program were enrolled, the current findings might not be generalizable to all institutionalized older adults with MNCD. Fourth, due to the nature of the exergame which adapts the training stimulus to the individual level of the participant, we did not evaluate a standardized protocol that would have allowed us to explore a dose-response relation in a more rigorous way. The observed training intensities varied from mild to moderate. Fifth, women were over-represented (77.8%). This over-representation is however due to the fact that women are at greater risk for developing Alzheimer’s Disease [93] and the number of women living in long-term care facilities in Belgium is higher [94]. Sixth, no long-term follow-up was conducted. Seventh, we did not include a physically active control condition such as a combined aerobic, balance, and strength training program. Eighth, we did not control for the effect of concurrent medication use including for example cholinesterase inhibitors, memantine, typical and atypical antipsychotics, antidepressants, and benzodiazepines, although it is known they may act as potential confounders and disruptors in MNCD trials [95].

## Conclusion

In conclusion, the present pilot RCT demonstrates that an individually adapted exergame training program improves lower extremity functioning, cognitive function, and step reaction time and reduces symptoms of depression in a sample of multi-morbid older adults with MNCD. Moreover, the adherence rates and lack of adverse events indicate that exergaming is an effective strategy for motivating people with MNCD dwelling in a nursing home to be physically active.

## Supplementary Information

**Additional file 1.**

**Additional file 2.**

**Additional file 3.**

**Additional file 4.**

## Data Availability

The datasets generated and/or analyzed during the current study are available from the corresponding author on reasonable request.

## Abbreviations

ADL: Activities of daily living
ANCOVA: Analysis of covariance
BBS: Berg Balance Scale
CONSORT: Consolidated Standards of Reporting Trials
CSDD: Cornell scale for depression in dementia
DQoL: Dementia quality of life
MMSE: Mini-Mental State Examination
MoCA: Montréal Cognitive Assessment
NPI: Neuropsychiatric inventory
RCT: Randomized controlled trial
SPPB: Short Physical Performance Battery
SRTT: Step reaction time test
TIDieR: Template for Intervention Description and Replication
UZ Leuven: Universitair Ziekenhuis Leuven
USB: Universal serial bus

## References

[1] LoGiudice D, Watson R (2014). Dementia in older people: an update. Intern Med J.

[2] Alzheimer’s Disease International (2019). World Alzheimer Report 2019: attitudes to dementia.

[3] Arvanitakis Z, Shah RC, Bennett DA (2019). Diagnosis and management of dementia: review. Jama..

[4] World Alzheimer Report 2016 | Alzheimer’s Disease International 2016 [updated 2016-09-20. Available from: https://www.alz.co.uk/research/world-report-2016. Accessed 24 Oct 2020.

[5] WHO. Dementia: a public health priority. WHO. 2016.

[6] Tisher A, Salardini A (2019). A comprehensive update on treatment of dementia. Semin Neurol.

[7] Vancampfort D, Solmi M, Firth J, Vandenbulcke M, Stubbs B (2020). The impact of pharmacologic and nonpharmacologic interventions to improve physical health outcomes in people with dementia: a meta-review of meta-analyses of randomized controlled trials. J Am Med Dir Assoc.

[8] Anderiesen H, Scherder E, Goossens R, Sonneveld M (2014). A systematic review - physical activity in dementia: the influence of the nursing home environment. Appl Ergon.

[9] Forbes D, Forbes SC, Blake CM, Thiessen EJ, Forbes S. Exercise programs for people with dementia. Cochrane Database Syst Rev. 2015;(4):CD006489. 10.1002/14651858.CD006489.pub4. 10.1002/14651858.CD006489.pub4 PMC9426996 25874613

[10] Karssemeijer EGA, Aaronson JA, Bossers WJ, Smits T, Olde Rikkert MGM, Kessels RPC (2017). Positive effects of combined cognitive and physical exercise training on cognitive function in older adults with mild cognitive impairment or dementia: a meta-analysis. Ageing Res Rev.

[11] Groot C, Hooghiemstra AM, Raijmakers PG, van Berckel BN, Scheltens P, Scherder EJ (2016). The effect of physical activity on cognitive function in patients with dementia: a meta-analysis of randomized control trials. Ageing Res Rev.

[12] Dyer AH, Lawlor B, Kennelly SP (2020). Gait speed, cognition and falls in people living with mild-to-moderate Alzheimer disease: data from NILVAD. BMC Geriatr.

[13] van Doorn C, Gruber-Baldini AL, Zimmerman S, Hebel JR, Port CL, Baumgarten M (2003). Dementia as a risk factor for falls and fall injuries among nursing home residents. J Am Geriatr Soc.

[14] Kolanowski AM, Litaker M, Buettner L (2005). Efficacy of theory-based activities for behavioral symptoms of dementia. Nurs Res.

[15] van Santen J, Droes RM, Holstege M, Henkemans OB, van Rijn A, de Vries R (2018). Effects of exergaming in people with dementia: results of a systematic literature review. J Alzheimers Dis.

[16] Ikezoe T, Asakawa Y, Shima H, Kishibuchi K, Ichihashi N (2013). Daytime physical activity patterns and physical fitness in institutionalized elderly women: an exploratory study. Arch Gerontol Geriatr.

[17] Dove E, Astell AJ (2017). The use of motion-based technology for people living with dementia or mild cognitive impairment: a literature review. J Med Internet Res.

[18] Kappen DL, Mirza-Babaei P, Nacke LE (2019). Older adults’ physical activity and exergames: a systematic review. Int J Hum Comput Interact.

[19] Stanmore E, Stubbs B, Vancampfort D, de Bruin ED, Firth J (2017). The effect of active video games on cognitive functioning in clinical and non-clinical populations: a meta-analysis of randomized controlled trials. Neurosci Biobehav Rev.

[20] Kathrin G, Regan M (2014). Custom-designed motion-based games for older adults: a review of literature in human-computer interaction. Gerontechnology.

[21] Swinnen N, Vandenbulcke M, de Bruin ED, Akkerman R, Stubbs B, Vancampfort D. Exergaming for people with major neurocognitive disorder: a qualitative study. Disabil Rehabil. 2020:1–9. 10.1080/09638288.2020.1822934. 10.1080/09638288.2020.1822934 32962436

[22] Dietlein C, Eichberg S, Fleiner T, Zijlstra W (2018). Feasibility and effects of serious games for people with dementia: a systematic review and recommendations for future research. Gerontechnology..

[23] McCallum S, Boletsis C. A taxonomy of serious games for dementia. Games Health. 2013:219–32. 10.1007/978-3-658-02897-8_17.

[24] Swinnen N, Vandenbulcke M, Vancampfort D. Exergames in people with major neurocognitive disorder: a systematic review. Disabil Rehabil Assist Technol. 2020. p. 1–14. 10.1080/17483107.2020.1785566. 10.1080/17483107.2020.1785566 32697614

[25] Karssemeijer EGA, Aaronson JA, Bossers WJR, Donders R, Olde Rikkert MGM, Kessels RPC (2019). The quest for synergy between physical exercise and cognitive stimulation via exergaming in people with dementia: a randomized controlled trial. Alzheimers Res Ther.

[26] Karssemeijer EGA, Bossers WJR, Aaronson JA, Sanders LMJ, Kessels RPC, Olde Rikkert MGM (2019). Exergaming as a physical exercise strategy reduces frailty in people with dementia: a randomized controlled trial. J Am Med Dir Assoc.

[27] Eldridge SM, Chan CL, Campbell MJ, Bond CM, Hopewell S, Thabane L (2016). CONSORT 2010 statement: extension to randomised pilot and feasibility trials. BMJ..

[28] Guralnik JM, Simonsick EM, Ferrucci L, Glynn RJ, Berkman LF, Blazer DG, Scherr PA, Wallace RB (1994). A short physical performance battery assessing lower extremity function: association with self-reported disability and prediction of mortality and nursing home admission. J Gerontol.

[29] Fox B, Henwood T, Neville C, Keogh J (2014). Relative and absolute reliability of functional performance measures for adults with dementia living in residential aged care. Int Psychogeriatr.

[30] Julayanont P, Phillips N, Chertkow H, Nasreddine ZS. Montreal Cognitive Assessment (MoCA): concept and clinical review. Cognitive screening instruments: Springer; 2013. p. 111–51. DOI: 10.1007/978-1-4471-2452-8_6.

[31] Nasreddine ZS, Phillips NA, Bédirian V, Charbonneau S, Whitehead V, Collin I (2005). The Montreal Cognitive Assessment, MoCA: a brief screening tool for mild cognitive impairment. J Am Geriatr Soc.

[32] Koski L, Xie H, Konsztowicz S (2011). Improving precision in the quantification of cognition using the Montreal Cognitive Assessment and the Mini-Mental State Examination. Int Psychogeriatr.

[33] Cummings JL (1997). The Neuropsychiatric Inventory: assessing psychopathology in dementia patients. Neurology..

[34] Alexopoulos GS, Abrams RC, Young RC, Shamoian CA (1988). Cornell scale for depression in dementia. Biol Psychiatry.

[35] Wolak-Thierry A, Novella JL, Barbe C, Morrone I, Mahmoudi R, Jolly D (2015). Comparison of QoL-AD and DQoL in elderly with Alzheimer’s disease. Aging Ment Health.

[36] Brod M, Stewart AL, Sands L, Walton P (1999). Conceptualization and measurement of quality of life in dementia: the dementia quality of life instrument (DQoL). Gerontologist..

[37] Roberts RO, Knopman DS, Mielke MM, Cha RH, Pankratz VS, Christianson TJH, Geda YE, Boeve BF, Ivnik RJ, Tangalos EG, Rocca WA, Petersen RC (2014). Higher risk of progression to dementia in mild cognitive impairment cases who revert to normal. Neurology..

[38] Katz S, Ford AB, Moskowitz RW, Jackson BA, Jaffe MW (1963). Studies of illness in the aged: the index of ADL: a standardized measure of biological and psychosocial function. Jama..

[39] Liao YY, Chen IH, Wang RY (2019). Effects of Kinect-based exergaming on frailty status and physical performance in prefrail and frail elderly: a randomized controlled trial. Sci Rep.

[40] Kendzierski D, DeCarlo KJ (1991). Physical activity enjoyment scale: two validation studies. J Sport Exerc Psychol.

[41] Goldberg A, Schepens S (2011). Measurement error and minimum detectable change in 4-meter gait speed in older adults. Aging Clin Exp Res.

[42] Kuys SS, Peel NM, Klein K, Slater A, Hubbard RE (2014). Gait speed in ambulant older people in long term care: a systematic review and meta-analysis. J Am Med Dir Assoc.

[43] Menz HB, Lord SR, Fitzpatrick RC (2003). Age-related differences in walking stability. Age Ageing.

[44] Tiedemann A, Sherrington C, Lord SR (2005). Physiological and psychological predictors of walking speed in older community-dwelling people. Gerontology..

[45] Whitehead AL, Julious SA, Cooper CL, Campbell MJ (2016). Estimating the sample size for a pilot randomised trial to minimise the overall trial sample size for the external pilot and main trial for a continuous outcome variable. Stat Methods Med Res.

[46] Nyman SR, Victor CR (2011). Older people’s recruitment, sustained participation, and adherence to falls prevention interventions in institutional settings: a supplement to the Cochrane systematic review. Age Ageing.

[47] Trivedi D (2017). Cochrane review summary: Mini-Mental State Examination (MMSE) for the detection of dementia in clinically unevaluated people aged 65 and over in community and primary care populations. Prim Health Care Res Dev.

[48] Wiloth S, Lemke N, Werner C, Hauer K (2016). Validation of a computerized, game-based assessment strategy to measure training effects on motor-cognitive functions in people with dementia. JMIR Serious Games.

[49] Wiloth S, Werner C, Lemke NC, Bauer J, Hauer K. Motor-cognitive effects of a computerized game-based training method in people with dementia: a randomized controlled trial. Aging Ment Health. 2018;22(9):1124–35. 10.1080/13607863.2017.1348472. Epub 2017 Jul 6. 10.1080/13607863.2017.1348472 28682124

[50] Wittelsberger R, Krug S, Tittlbach S, Bos K (2013). The influence of Nintendo-Wii(R) bowling upon residents of retirement homes. Zeitschrift fur Gerontologie und Geriatrie.

[51] Healy AF, Kole JA, Bourne LE (2014). Training principles to advance expertise. Front Psychol.

[52] Hoffmann TC, Glasziou PP, Boutron I, Milne R, Perera R, Moher D, Altman DG, Barbour V, Macdonald H, Johnston M, Lamb SE, Dixon-Woods M, McCulloch P, Wyatt JC, Chan AW, Michie S (2014). Better reporting of interventions: template for intervention description and replication (TIDieR) checklist and guide. Bmj..

[53] Zhang Y, Cai J, An L, Hui F, Ren T, Ma H, Zhao Q (2017). Does music therapy enhance behavioral and cognitive function in elderly dementia patients? A systematic review and meta-analysis. Ageing Res Rev.

[54] Kim H-J, Park I, Lee HJ, Lee O (2016). The reliability and validity of gait speed with different walking pace and distances against general health, physical function, and chronic disease in aged adults. J Exerc Nutr Biochem.

[55] Olsen CF, Bergland A (2017). Reliability of the Norwegian version of the short physical performance battery in older people with and without dementia. BMC Geriatrics.

[56] Hauer K, Oster P (2008). Measuring functional performance in persons with dementia. J Am Geriatr Soc.

[57] Trumpf R, Morat T, Zijlstra W, Haussermann P, Fleiner T (2020). Assessment of functional performance in acute geriatric psychiatry – time for new strategies?. J Geriatr Psychiatry Neurol.

[58] Guralnik JM, Ferrucci L, Pieper CF, Leveille SG, Markides KS, Ostir GV, Studenski S, Berkman LF, Wallace RB (2000). Lower extremity function and subsequent disability: consistency across studies, predictive models, and value of gait speed alone compared with the short physical performance battery. J Gerontol A Biol Sci Med Sci.

[59] Ostir GV, Volpato S, Fried LP, Chaves P, Guralnik JM (2002). Reliability and sensitivity to change assessed for a summary measure of lower body function: results from the Women’s Health and Aging Study. J Clin Epidemiol.

[60] Lord SR, Fitzpatrick RC (2001). Choice stepping reaction time: a composite measure of falls risk in older people. J Gerontol A Biol Sci Med Sci.

[61] Delbaere K, Gschwind YJ, Sherrington C, Barraclough E, Garrués-Irisarri MA, Lord SR (2016). Validity and reliability of a simple ‘low-tech’ test for measuring choice stepping reaction time in older people. Clin Rehabil.

[62] Freitas S, Simões MR, Marôco J, Alves L, Santana I (2012). Construct validity of the Montreal Cognitive Assessment (MoCA). J Int Neuropsychol Soc.

[63] De Roeck EE, De Deyn PP, Dierckx E, Engelborghs S (2019). Brief cognitive screening instruments for early detection of Alzheimer’s disease: a systematic review. Alzheimers Res Ther.

[64] Connor DJ, Sabbagh MN, Cummings JL (2008). Comment on administration and scoring of the Neuropsychiatric Inventory in clinical trials. Alzheimers Dementia.

[65] Cummings JL (1994). The Neuropsychiatric Inventory.

[66] Barca ML, Barca B (2010). A reliability and validity study of the Cornell scale among elderly inpatients, using various clinical criteria. Dement Geriatr Cogn Disord.

[67] Ferretti-Rebustini RE, Balbinotti MA, Jacob-Filho W, Rebustini F, Suemoto CK, Pasqualucci CA, Farfel JM, Leite RE, Grinberg LT, Nitrini R (2015). Validity of the Katz Index to assess activities of daily living by informants in neuropathological studies. Rev Esc Enferm USP.

[68] de Bruin ED, Reith A, Dörflinger M, Murer K. Feasibility of strength-balance training extended with computer game dancing in older people; does it affect dual task costs of walking? J Novel Physiother. 2011;1:104. 10.4172/2165-7025.1000104.

[69] Sterne JAC, White IR, Carlin JB, Spratt M, Royston P, Kenward MG, Wood AM, Carpenter JR (2009). Multiple imputation for missing data in epidemiological and clinical research: potential and pitfalls. BMJ..

[70] Cohen (1988). Statistical power analysis for the behavioral sciences. 2nd edn.

[71] van Santen J, Dröes R-M, Twisk JWR, Blanson Henkemans OA, van Straten A, Meiland FJM (2020). Effects of exergaming on cognitive and social functioning of people with dementia: a randomized controlled trial. J Am Med Dir Assoc.

[72] Kappen DL, Mirza-Babaei P, Nacke LE (2018). Older adults’ physical activity and exergames: a systematic review. Int J Hum Comput Interact.

[73] Tahmosybayat R, Baker K, Godfrey A, Caplan N, Barry G (2018). Movements of older adults during exergaming interventions that are associated with the Systems Framework for Postural Control: a systematic review. Maturitas..

[74] Dodwell G, Muller HJ, Tollner T (2019). Electroencephalographic evidence for improved visual working memory performance during standing and exercise. Br J Psychol.

[75] Rosenbaum D, Mama Y, Algom D (2017). Stand by Your Stroop: standing up enhances selective attention and cognitive control. Psychol Sci.

[76] Padala KP, Padala PR, Malloy TR, Geske JA, Dubbert PM, Dennis RA (2012). Wii-fit for improving gait and balance in an assisted living facility: a pilot study. J Aging Res.

[77] Padala KP, Padala PR, Lensing SY, Dennis RA, Bopp MM, Roberson PK, Sullivan DH (2017). Home-based exercise program improves balance and fear of falling in community-dwelling older adults with mild Alzheimer’s disease: a pilot study. J Alzheimers Dis.

[78] Fanning J, Rejeski WJ, Chen SH, Guralnik J, Pahor M, Miller ME. Relationships between profiles of physical activity and major mobility disability in the LIFE study. J Am Geriatrics Soc. 2020;68(7):1476–83. 10.1111/jgs.16386. Epub 2020 Mar 20. 10.1111/jgs.16386 PMC8284920 32196636

[79] Penninx BW, Ferrucci L, Leveille SG, Rantanen T, Pahor M, Guralnik JM (2000). Lower extremity performance in nondisabled older persons as a predictor of subsequent hospitalization. J Gerontol A Biol Sci Med Sci.

[80] Fisher S, Ottenbacher KJ, Goodwin JS, Graham JE, Ostir GV (2009). Short physical performance battery in hospitalized older adults. Aging Clin Exp Res.

[81] Pavasini R, Guralnik J, Brown JC, di Bari M, Cesari M, Landi F, Vaes B, Legrand D, Verghese J, Wang C, Stenholm S, Ferrucci L, Lai JC, Bartes AA, Espaulella J, Ferrer M, Lim JY, Ensrud KE, Cawthon P, Turusheva A, Frolova E, Rolland Y, Lauwers V, Corsonello A, Kirk GD, Ferrari R, Volpato S, Campo G (2016). Short Physical Performance Battery and all-cause mortality: systematic review and meta-analysis. BMC Med.

[82] Lauretani F, Ticinesi A, Gionti L, Prati B, Nouvenne A, Tana C, Meschi T, Maggio M (2019). Short-Physical Performance Battery (SPPB) score is associated with falls in older outpatients. Aging Clin Exp Res.

[83] Drazich BF, LaFave S, Crane BM, Szanton SL, Carlson MC, Budhathoki C, Taylor JL (2020). Exergames and depressive symptoms in older adults: a systematic review. Games Health.

[84] Dove E, Astell A (2019). The Kinect Project: group motion-based gaming for people living with dementia. Dementia (London).

[85] Wang R, Holsinger RMD (2018). Exercise-induced brain-derived neurotrophic factor expression: therapeutic implications for Alzheimer’s dementia. Ageing Res Rev.

[86] Eggenberger P, Wolf M, Schumann M, de Bruin ED (2016). Exergame and balance training modulate prefrontal brain activity during walking and enhance executive function in older adults. Front Aging Neurosci.

[87] Berry AS, Zanto TP, Clapp WC, Hardy JL, Delahunt PB, Mahncke HW, Gazzaley A (2010). The influence of perceptual training on working memory in older adults. PLoS One.

[88] Kraft E (2012). Cognitive function, physical activity, and aging: possible biological links and implications for multimodal interventions. Aging Neuropsychol Cognit.

[89] Gheysen F, Poppe L, DeSmet A, Swinnen S, Cardon G, De Bourdeaudhuij I (2018). Physical activity to improve cognition in older adults: can physical activity programs enriched with cognitive challenges enhance the effects? A systematic review and meta-analysis. Int J Behav Nutr Phys Act.

[90] Fissler P, Küster O, Schlee W, Kolassa I-T. Chapter 16 - Novelty Interventions to Enhance Broad Cognitive Abilities and Prevent Dementia: Synergistic Approaches for the Facilitation of Positive Plastic Change. In: Merzenich MM, Nahum M, Van Vleet TM, editors. Progress in Brain Research. 207: Elsevier; 2013. 403–34. 10.1016/B978-0-444-63327-9.00017-5 24309264

[91] Okubo Y, Schoene D, Lord SR (2017). Step training improves reaction time, gait and balance and reduces falls in older people: a systematic review and meta-analysis. Br J Sports Med.

[92] van Santen J, Dröes RM, Bosmans JE, Blanson Henkemans OA, van Bommel S, Hakvoort E, Valk R, Scholten C, Wiersinga J, van Straten A, Meiland F (2019). The (cost-) effectiveness of exergaming in people living with dementia and their informal caregivers: protocol for a randomized controlled trial. BMC Geriatr.

[93] Podcasy JL, Epperson CN (2016). Considering sex and gender in Alzheimer disease and other dementias. Dialogues Clin Neurosci.

[94] overheid V. Statistiek Vlaanderen. Care and assistance for elderly people 2018 [Available from: https://www.statistiekvlaanderen.be/en/care-and-assistance-for-elderly-people. Accessed 19 Feb 2021.

[95] Liyanage SI, Santos C, Weaver DF (2018). The hidden variables problem in Alzheimer’s disease clinical trial design. Alzheimers Dement (N Y).

