# Supplementary material for: The efficacy of exergaming in people with major neurocognitive disorder residing in long-term care facilities: a pilot randomized controlled trial
Source: Alzheimers Res Ther. 2021 Mar 30;13:70. doi: 10.1186/s13195-021-00806-7 (PMC8008333; doi:10.1186/s13195-021-00806-7)
Supplement: Supplementary file 1 — Additional file 1. [file 13195_2021_806_MOESM1_ESM.docx]

**Additional file 1.** CONSORT diagram of participant flow

Allocated to control; music (*n* = 27)

Allocated to intervention; exergame (*n* = 28)

Randomized (*n* = 55)

Assessed for eligibility (*n* = 114)

## Enrollment

Excluded (*n* = 59)

♦  Not meeting inclusion criteria (*n* = 44)

♦  Declined to participate (*n* = 15)

## Allocation

## Post intervention

Completed control (*n* = 22)
Discontinued intervention due to transfer (*n* = 2)
Discontinued intervention due to Covid-19 measures (*n* = 3)

3)

(give reasons) (*n* = )

Completed intervention (*n* = 23)
Discontinued intervention due to transfer (*n* = 3)
Discontinued intervention due to Covid-19 measures (*n* = 2)

Analyzed (*n* = 22)

Analyzed (*n* = 23)

## Analysis
