# Supplementary material for: The efficacy of exergaming in people with major neurocognitive disorder residing in long-term care facilities: a pilot randomized controlled trial
Source: Alzheimers Res Ther. 2021 Mar 30;13:70. doi: 10.1186/s13195-021-00806-7 (PMC8008333; doi:10.1186/s13195-021-00806-7)
Supplement: Supplementary file 2 — Additional file 2. [file 13195_2021_806_MOESM2_ESM.docx]

**Additional file 2.** The Template for Intervention Description and Replication (TIDieR) Checklist

| Item number | Item | Located where |
| --- | --- | --- |
|  | **BRIEF NAME** |  |
| **1.** | Standing exergaming program in people with MNCD residing in long-term care facilities. |  |
|  | **WHY** |  |
| **2.** | Exergames are innovative developments that combine physical activity and cognitive tasks. It is a motivating strategy to engage people to be physically active. The efficacy of a standing exergame program on physical fitness parameters including mobility and balance, neuropsychiatric symptoms, mood, and quality of life should be investigated in people with MNCD residing in long-term care facilities. |  |
|  | **WHAT** |  |
| **3.** | **Materials**: The exergame group performed exergaming on the Dividat Senso device (dividat.com/en/senso). The control group watched preferred music videos (youtube.com). |  |
| **4.** | **Procedures**: The exergame intervention group performed three times weekly for eight weeks 15 minutes of exergaming. The music control group watched preferred music videos three times weekly for eight weeks 15 minutes. Both groups were supervised by a physical therapist and both the interventions were added to care as usual. |  |
|  | **WHO PROVIDED** |  |
| **5.** | Participants from the exergame intervention and music control group were supervised and assessed by a physical therapist who had five years’ experience in working with people with MNCD. |  |
|  | **HOW** |  |
| **6.** | Participants from the exergame intervention and music control group were individually supervised face-to-face and individually assessed face-to-face at baseline (pretest) and after eight weeks (posttest). |  |
|  | **WHERE** |  |
| **7.** | Inpatients of the University Psychiatric Centre KU Leuven campus Kortenberg (Vlaams Brabant, Belgium) performed the exergames and music sessions in a separate room in the facility. The assessments also took place in this room. The NPI and CSDD were assessed in the form of an interview with the caregiver and took place in a meeting room.  Residents of long-term care facility de Wingerd in Leuven (Vlaams Brabant, Belgium) performed the exergames and music sessions in an exercise room in the facility. The assessments also took place in this room. The NPI and CSDD were assessed in the form of an interview with the caregiver and took place in the living unit of the residence. |  |
|  | **WHEN and HOW MUCH** |  |
| **8.** | Participants were included after residing at least two weeks in the care facility. They performed three individual sessions per week for a period of eight weeks, resulting in a total of maximum 24 sessions. Each session consisted of a walk to the exercise room (i.e. approximately 10 minutes), 15 minutes of exergaming and a walk back to the ward. |  |
|  | **TAILORING** |  |
| **9.** | The physical therapist designed an individual exergame program for each participant, adapted to the participants’ functionality, cognition, and health status. The exergames automatically adapted to the participants’ capabilities during active exergaming, i.e., providing more difficult stimuli when the players reacted fast and correct. During the 8-week program, progress was also made regarding the reduction of manual support via the bars. |  |
|  | **MODIFICATIONS** |  |
| **10.^ǂ^** | The study ended prematurely due to Covid-19 measures. |  |
|  | **HOW WELL** |  |
| **11.** | Caregivers were informed about the study and they were kept up-to-date verbally during the course of the intervention.  There was a close collaboration between the physical therapist and the other involved investigators.  Pre- and post- outcomes were discussed concisely with the participant after completing the intervention and feedback was given to the caregivers in an information sheet that was added to the patient or resident files. Family members who wished to be contacted after the trial were also provided with feedback. |  |
| **12.^ǂ^** | Attendance sheets were filled in during each session to record the number of training sessions. Attendance rates were measured by calculating the percentage of the attended training sessions, divided by the maximum possible training sessions (24 sessions). The mean attendance rate was 82.9% in the exergame intervention group and 73.7% in the music control group. There were no study-related adverse events reported by the participants, nor observed by the research team. |  |

CSDD: Cornell Scale for Depression in Dementia; MNCD: Major NeuroCognitive Disorder; NPI: Neuropsychiatric Inventory
