# Supplementary material for: The efficacy of exergaming in people with major neurocognitive disorder residing in long-term care facilities: a pilot randomized controlled trial
Source: Alzheimers Res Ther. 2021 Mar 30;13:70. doi: 10.1186/s13195-021-00806-7 (PMC8008333; doi:10.1186/s13195-021-00806-7)
Supplement: Supplementary file 3 — Additional file 3. [file 13195_2021_806_MOESM3_ESM.docx]

**Additional file 3.** Description of the Video Games

*Simple*

In the Simple game, four white circles were displayed on a blue background on the television screen. These circles corresponded to the four arrows on the dance platform (up, down, left and right). Each time a circle turned red, the participant had to take a step in the corresponding plane on the platform. The program was designed so that the speed accelerated in line with game performance.
The Simple game trained focused attention, i.e. the ability to concentrate on stimuli. It also trained the psychomotor processing speed and responsiveness, i.e. process and react to sensory stimuli as quickly as possible.

*Birds*

The second video game was called Birds*.* A feather of a certain colour (blue, green, orange, yellow or red) was surrounded by four different objects. One of the objects was a bird that corresponded with the colour of the feather. The other objects were a bee, a flower, a beetle etc. The participant had to place a foot in the plane which corresponded with the bird. If the participant’s step was correct, a chirping sound was heard.

The Birds game trained selective attention, i.e. the ability to react to certain (relevant) stimuli and to be able to ignore irrelevant stimuli.

*Targets*

During the third video game called Targets, four targets were displayed on the television screen. These four targets corresponded to the four planes on the pressure platform. Multiple black balls appeared from and to different directions on the screen and passed one of the four targets. The participant had to place one foot in the plane which corresponded to the target along which the ball passed. When the participant’s foot pushed the right plane at the right moment, i.e. the moment when the ball passed the target, the ball turned green and a positive sound was played. The main goal was to achieve as many balls as possible to turn from black to green. The program has been designed so that the balls appeared a little faster each time and with an increasing amount. In that way, the video game became more challenging, but when it became too difficult, it immediately adapted to the maximal achievable level of the participant.

The Targets game trained forward thinking and targeted reaction with correct timing.

Depending on the cognitive status and physical abilities of the participants, one or more of these three video games were played. In most of the cases, the intervention was started with Simple and Birds and if the participant was able to perform them properly, Targets was also introduced.

In some cases, progress was made to the more complex games Seasons and Habitats. Seasons and Habitats are similar games but have different audio-visual designs. This was based on the cognitive abilities of the participant and in consultation with the participant.

The Habitats game consisted of four parts on the screen, each representing one of the four seasons. During the video game, objects related to one of the four seasons appeared and passed along one of the four parts of the screen. If the object did not match the season, the participant had to place a foot in the plane that represented the season. If stepped correct, positive visual feedback was shown on the screen.

The Habitats game trained selective attention, i.e. the ability to react to certain stimuli and to be able to ignore other stimuli. Provoked reactions are to be suppressed by the player.

The games and the sequences were adapted individually in all cases, so the training programs were not standardised.
