# Supplementary material for: The efficacy of exergaming in people with major neurocognitive disorder residing in long-term care facilities: a pilot randomized controlled trial
Source: Alzheimers Res Ther. 2021 Mar 30;13:70. doi: 10.1186/s13195-021-00806-7 (PMC8008333; doi:10.1186/s13195-021-00806-7)
Supplement: Supplementary file 4 — Additional file 4. [file 13195_2021_806_MOESM4_ESM.docx]

| **Variable** | **Intervention (n= 23)** | | **Control (n=22)** | | **P** | **F** | ***η²_p_*** |
| --- | --- | --- | --- | --- | --- | --- | --- |
|  | Pre test  Median (IQR) | Post test  Median (IQR) | Pre test  Median (IQR) | Post test  Median (IQR) |  |  |  |
| SPPB | 5.0 (2.0) | 9.0 (4.0) | 5.0 (4.0) | 3.0 (2.0) | <0.001* | 72.1 | 0.64 |
| Gait speed (m/s) | () | () | () | () | <0.001* | 29.3 | 0.41 |
| SRTT (ms) | 2486.1 (2921.9) | 1376.9 (545.4) | 2915.6 (3061.2) | 3491.3 (4424.4) | <0.001* | 38.8 | 0.51 |
| MoCA | 8.0 (9.0) | 12.0 (10.0) | 8.0 (8.0) | 5.0 (4.0) | <0.001* | 24.4 | 0.38 |
| NPI | 7.0 (22.2) | 3.0 (5.7) | 8.0 (11.0) | 12.0 (19.0) | 0.165 | 2.0 | 0.05 |
| CSDD | 7.0 (9.0) | 2.0 (3.0) | 5.0 (9.0) | 7.0 (10.0) | <0.001* | 28.8 | 0.43 |
| DQoL | 3.0 (1.0) | 3.5 (1.0) | 3.0 (0.0) | 2.0 (0.0) | 0.012 | 6.9 | 0.16 |
| ADL | 9.0 (4.0) | 9.0 (4.0) | 9.0 (4.0) | 10.0 (6.0) | 0.008 | 7.7 | 0.16 |

**Additional file 4: Supplementary Table 1.** The effects of an exergame intervention and a music intervention on measured outcomes

* Significant when P < 0.00625 (0.05 / 8 comparisons) using Quade’s non-parametric analyses of covariance with post test scores as dependent variables, groups as independent variables, and baseline scores as covariates. ADL: Activities of daily living (range = 6 to 24 with higher scores indicating higher dependency in activities of daily living); CSDD: Cornell scale for depression in dementia (range = 0 to 38, a score below 6 is associated with absence of depressive symptoms, and scores above 10 indicate probable major depression); DQoL: Dementia quality of life (scores range from 1 (poor QoL) to 5 (excellent QoL)); IQR: interquartile range; MoCA: Montréal cognitive assessment (total scores range from 0 to 30 with lower scores indicating more cognitive impairment); NPI: neuropsychiatric inventory (12 item score with a range of 0 to 12 per item); SPPB: short physical performance battery (total scores range from 0 to 12 with lower scores indicating a higher risk and a score lower than 10 indicates one or more mobility limitations); SRTT: step reaction time test (lower values indicate a faster reaction time)
